# The Dual Role of MicroRNAs in Colorectal Cancer Progression

**DOI:** 10.3390/ijms19092791

**Published:** 2018-09-17

**Authors:** Lei Ding, Zhenwei Lan, Xianhui Xiong, Hongshun Ao, Yingting Feng, Huan Gu, Min Yu, Qinghua Cui

**Affiliations:** 1 Lab of Biochemistry & Molecular Biology, School of Life Sciences, Yunnan University, Kunming 650091, China; dingleiynu@ynu.edu.cn (L.D.); lanzhenweiL@163.com (Z.L.); xiongxianhui1995@163.com (X.X.); aohongshun@163.com (H.A.); fengyingting1994@163.com (Y.F.); albertwesker0112@163.com (H.G.); yumin@ynu.edu.cn (M.Y.); 2 Key Lab of Molecular Cancer Biology, Yunnan Education Department, Kunming 650091, China

**Keywords:** colorectal cancer (CRC), oncogenic miRNAs, tumor-suppressive miRNAs, biomarkers

## Abstract

Colorectal cancer (CRC) is responsible for one of the major cancer incidence and mortality worldwide. It is well known that MicroRNAs (miRNAs) play vital roles in maintaining the cell development and other physiological processes, as well as, the aberrant expression of numerous miRNAs involved in CRC progression. MiRNAs are a class of small, endogenous, non-coding, single-stranded RNAs that bind to the 3’-untranslated region (3′-UTR) complementary sequences of their target mRNA, resulting in mRNA degradation or inhibition of its translation as a post-transcriptional regulators. Moreover, miRNAs also can target the long non-coding RNA (lncRNA) to regulate the expression of its target genes involved in proliferation and metastasis of CRC. The functions of these dysregulated miRNAs appear to be context specific, with evidence of having a dual role in both oncogenes and tumor suppression depending on the cellular environment in which they are expressed. Therefore, the unique expression profiles of miRNAs relate to the diagnosis, prognosis, and therapeutic outcome in CRC. In this review, we focused on several oncogenic and tumor-suppressive miRNAs specific to CRC, and assess their functions to uncover the molecular mechanisms of tumor initiation and progression in CRC. These data promised that miRNAs can be used as early detection biomarkers and potential therapeutic target in CRC patients.

## 1. Introduction

Colorectal cancer (CRC) is the third leading cause of cancer related mortality in both men and women, with an incidence approaching over 1.4 million people and about 693,900 deaths annually [1]. Approximately 60% of CRC patients are diagnosed with localized or distant metastases, termed stage IV, with a 5-year survival rate ranging from 12.5% to 70.4%, and poor prognosis, compared to over 90% for stage I [2]. Therefore, these facts highlight the urgent need to develop early molecular biomarkers for CRC. CRC is a heterogeneous multifactorial disease, with approximately 35% of the CRC is attributed to genetic factors. About 50 associated loci have already been identified by genome-wide association studies [3]. Moreover, smoking, alcohol consumption, low physical activity, obesity, and environmental factors have been linked to increase CRC risk [4].

Currently, chemotherapy including anti-cancer drugs and compounds are used as the primary treatment in advanced stage of the disease, or as an adjuvant treatment after surgery in case of lymph node metastasis [5]. Surgery combined with chemotherapy and radiotherapy for most patients at stage III and IV is still suggested as the most effective approaches in treatment of CRC. However, these treatments are often associated with severe adverse reactions and chemo resistance [6].

5-fluorouracil (5-FU) is widely used for CRC therapy, which has been established as a first-line therapeutic agent for more than 50 years [7]. However, 5-FU is non-specific and CRC is resistant to it. Presently, adjuvant chemotherapy, such as 5-FU plus leucovorin (LV) (5-FU/LV), infusional 5-FU, LV, oxaliplatin (FOLFOX), tegafur plus uracil (UFT), or capecitabine have been developed and are now widely used against the CRC [8]. The key enzymes, such as thymidylate synthase (TS), thymidine phosphorylase (TP), orotate phosphoribosyltransferase (OPRT), and dihydropyrimidine dehydrogenase (DPD) were used as predictive biomarkers of the efficacy of 5-FU chemotherapy and targeted therapy in CRC cells [9]. Additionally, monoclonal antibodies were used as a therapy for CRC to target epidermal growth factor receptor (EGFR) and vascular endothelial growth factor (VEGF) [10,11]. However, many reports showed that the mutations of the target genes or the downstream signaling molecules greatly reduced their efficiency or even caused the clinical treatment to be inactivated [12].

## 2. MiRNA Biogenesis and Functions

MicroRNAs (miRNAs) significantly contribute to the initiation and development of various oncocytic molecular events, including tumor development, progression, and metastasis, which promised miRNAs as potential biomarkers for CRC progression and prognosis, with the hope of conjunction with conventional clinical parameters to gain more accurate diagnosis in CRC [13]. In this review, we will summarize the pathophysiological roles of miRNAs on survival rates and response to systemic chemotherapy in CRC, point to their potential use as diagnostic and prognostic biomarkers, focusing on their regulatory roles as a tumor suppressors and oncogenes in CRC.

MiRNAs are small (18–24 nucleotide), single-stranded, evolutionarily-conserved, non-coding RNAs that represent a class of endogenously-expressed small RNAs. The biogenesis of miRNAs involves a complex process including multiple stages. The nucleotides 2–7 of the mature miRNA have been identified as the most essential region for target mRNA recognition by base-pair complementary. The pre-miRNAs are exported to the cytoplasm by Exportin-5. RNase III endoribonuclease Dicer and its partner human immunodeficiency virus type 1 (HIV-1) transactivate response RNA binding protein (TRBP) guide pre-miRNAs into mature miRNAs composed of 20–24 nucleotides in the cytoplasm, which subsequently constitute the RNA-induced silencing complex (RISC) [14]. The targeted encoding proteins range from signaling proteins and transcription factors to RNA binding proteins with negative effects by directly binding to the 3’-untranslated region (3′-UTR) of target genes, which function as posttranscriptional regulators of gene expression through the repression of mRNA translation or degradation of target mRNA [15]. For instance, miR-16 promoted ovarian granulosa cell (GC) proliferation and inhibited apoptosis through directly targeting programmed cell death protein 4 (PDCD4) in polycystic ovarian syndrome (PCOS) [16]. Low expression of miR-577 is related to hepatic cellular cancer (HCC) growth through down-regulating β-catenin [17]. MiR-33a targets twist family basic helix-loop-helix transcription factor 1 (TWIST1) and inhibits invasion and metastasis in non-small cell lung cancer (NSCLC) [18]. Ectopic expression of miR-19a-3p contributes to HCC metastasis and chemo resistance by modulating phosphatase and tensin homolog (PTEN) expression and the PTEN-dependent pathways [19]. In addition, some study indicated the long non-coding RNA (lncRNA) is also the target of the miRNAs. For example, lncRNA overexpressed in colorectal cancer (OECC) directly targeted by miR-143-3p, led to down-regulation of gene nuclear factor κB (NF-κB) and p38 mitogen-activated protein kinase (MAPK) to promote CRC development [20]. LncRNA differentiation antagonizing nonprotein coding RNA (DANCR) and heat shock protein 27 (HSP27) are the targets of miR-577, they share the same binding sites, and their overexpression accelerates CRC proliferation and metastasis [21].

## 3. The Dual Role of miRNA in CRC

Multiple studies provide a concept regarding the roles of miRNA involved in colorectal carcinogenesis based on results from CRC tissues or cell lines. It is accepted that the involvement of miRNA in CRC appears to be context specific, with the evidence indicating that miRNAs have a dual role in tumor-suppressing and tumor-promoting activities [22].

### 3.1. Oncogenic miRNAs in CRC

Oncogenic miRNAs, also commonly termed as oncomiRs, mainly target and inhibit the expression of endogenous tumor-suppressor genes that accelerate carcinogenesis. The up-regulation of oncomiRs has a significant effect on the progression of CRC. Some current miRNAs appear to interfere with normal activity of important gene functions as oncogenes associated with CRC (Table 1).

MiR-21 is up-regulated in many types of human solid cancers. It is most highly up-regulated in CRC, targeted and down-regulated many genes, such as PDCD4, T-cell lymphoma invasion and metastasis 1 (TIAM1), sprouty homolog 2 (SPRY2), PTEN, transforming growth factor beta receptor II (TGFBR2), and cell division cycle 25A (CDC25A), which are involved in controlling proliferation, apoptosis, invasion, migration, and cancer stem cell (CSC) maintenance [23]. MiR21 functions as an oncomiR down-regulating the expression of PTEN through the PTEN/PI-3K/Akt signaling pathway in human colorectal cancer cells [24]. Asangani et al. reported that miR-21, via a specific binding site at nt228-249 of the 3′-UTR of PDCD4, negatively down-regulates tumor suppressor PDCD4 and stimulates invasion, intravasation, and metastasis in colorectal cancer [25]. Moreover, the high expression of miR-21 was associated with the resistance to chemotherapy of 5-FU. Valeri et al. demonstrated that the expression of miR-21 directly targeted the 3′-UTR of tumor suppressor human DNA MutS homolog 2 (hMSH2) and down-regulated its expression significantly, following dramatically reduced 5-FU-induced G2/M damage arrest and apoptosis [26]. Deng et al. further proved that miR-21 overexpression enhanced cell proliferation and invasion, inhibited apoptosis against the treatment of chemotherapeutic agent 5-FU in a HT29 CRC cell, and vice versa [27]. All of the results suggested that miR-21 could be used as a noninvasive potential biomarker for diagnostic and prognosis for CRC.

MiR-92a is a member of the known oncomiR cluster miR-17-92 located at chromosome 13q13, a region that frequently promotes cell proliferation, suppresses apoptosis of cancer cells, induces tumor angiogenesis, and accelerates tumor progression in CRC, lung cancers, hepatocellular carcinoma, esophageal squamous cell carcinoma, breast cancer, and stomach cancer [28]. In CRC, miR-92a, significantly up-regulated in the tissues or cell lines, led to down-regulation of E-cadherin and up-regulation of β-catenin and vimentin, involved in the regulation of epithelial-to-mesenchymal transition (EMT) via an interaction with its target, PTEN, through the PTEN/PI3K/Akt pathway [29]. Moreover, Nishida et al. reported that an association between high levels of miR-92a and lymphatic invasion, venous invasion and liver metastases through the pivotal genes, drosophila mothers against decapentaplegic protein (SMAD) family member 2 (SMAD2), SMAD4, and TGFBR2 of transforming growth factor beta (TGF-β) pathway based on the expression of miR-92a carcinoma cells and cancer-associated stroma compared with corresponding normal tissues [30]. The above results indicated that the high expression of miR-92a in CRC patients was associated with poor survival and miR-92a may be a potential screening biomarker for CRC diagnosis. Especially, the up-regulated expression level of miR-92a in stool or plasma has been shown to have a high specificity and sensitivity in predicting CRC or distinguish between CRC and healthy controls [31].

MiR-96 was found up-regulated in CRC samples and correlated with liver metastasis, which promotes the cell growth and proliferation via direct target p53 inducible nuclear protein 1 (TP53INP1) to down-regulate the activity of p53, forkhead box protein O1 (FOXO1) and FOXO3a in CRC [32]. In addition, miR-96 also plays a drug-sensitizing role via deregulating the anti-apoptotic regulator X-linked inhibitor of apoptosis (XIAP) and the p53 stability regulator ubiquitin-conjugating enzyme E2N (UBE2N), resulting in a synergy with cell-cycle-dependent drugs 5-FU and apoptotic cell death in CRC cells [33]. MiR-96 has been shown to promote cellular sensitivity to cisplatin and poly (ADP-ribose) polymerase (PARP) inhibition by down-regulating REV1 DNA-directed polymerase and RAD51 recombinase [34].

The miR-135 family, including two isoforms miR-135a and miR-135b, is highly conserved among mammals. MiR-135a and miR-135b were considered as an oncomiR in CRC. There are two copies of miR-135a in the human genome, the first copy is located in the first intron of the stabilin 1 (STAB1) gene on 3p21, and the second copy is located on 12q23 in intron 5 of the rhabdomyosarcoma 2-associated transcript (RMST) gene. However, the two genes have not been reported to be aberrant expressed in CRC. MiR-135b was located on 1q32.1 in the first intron of the LEM domain containing 1 (LEMD1) gene. Interestingly, the expression of this gene is up-regulated in CRC compared to the normal tissues [35] and the expression pattern of miR-135b is followed by the host gene and elevated by the same mechanism. Moreover, miR-135b often showed DNA copy number gain and the gene dosage effects were observed in CRC progression [36]. The up-regulation of miR-135 commonly occurs in CRC, with the concomitant down-regulation of adenomatous polyposis coli (APC), a key component of the Wnt signaling pathway and a precursor of CRC, via targeting the 3′-UTR of APC and inducing downstream Wnt pathway activity even when APC mutations are present. Additionally, miR-135 gene family as a regulator of APC seems specific and conserved in human [37]. The expression of miR-135b was inversely correlated with serum estradiol (E_2_) and estrogen receptor-β (ER-β) mRNA in CRC, affected DNA mismatch repair (MMR) system gene MutL homolog 1 (hMLH1) and hMSH2 [38]. Several studies reported the circulating miR-135 in plasma as a CRC biomarker for clinical stage analysis in CRC [39].

MiR-155, as one of the most salient oncogenic microRNA, is over-expressed in CRC, breast and lung cancer, mediating cell growth, invasion, migration, stemness, and angiogenesis [40]. High expression of miR-155 correlated with poor prognosis, drug resistance and genome instability in CRC patients [41]. MiR-155 directly binds to the 3’-UTR of protein tyrosine phosphatase, receptor type J (PTPRJ) mRNA to suppress the expression of PTPRJ through miR-155/PTPRJ/AKT axis, and affect the proliferation and migration of CRC cells [42]. Gironella et al. indicated that TP53INP1 is one of the target genes of miR-155 involved in the progression of adenocarcinomas of the stomach and colon [43]. Valeri et al. suggested that miR-155 played a vital role in the modulation of the mismatch repair (MMR) system via down-regulating MSH2, MSH6, and MLH1, inducing a mutator phenotype and microsatellite instability (MSI) [44]. Recent studies have demonstrated that a positive feedback loop that linked miR-155 with the transcription factor NF-κB, inhibition of miR-155 additionally enhanced the sensitivity of CRC cells to 5-FU chemotherapy [45], miR-155 and its target gene FOXO3a acquired radio-resistance via the PI3K/Akt pathway in CRC cell line, which promised miR-155 as a novel specific biomarker to distinguish radio-sensitive from radio-resistant CRC tumors [46]. In addition, research reported that miR-155 has an effect on CRC invasion metastasis. Ulivi et al. pointed out circulating plasma levels of miR-155 as predictors of bevacizumab efficacy in patients with metastatic CRC, which indicated that miR-155 could be a bio-marker in the treatment used concurrently with bevacizumab [47]. Zhang et al. confirmed that up-regulation of miR-155 plays an important role in promoting CRC cell migration and invasion, and acts as a mediator of EMT, through the regulation of claudin-1 expression [48]. Al-Haidari et al. reported that miR-155-5p controls colon cancer cell migration via post-transcriptional regulation of human antigen R (HuR) [49]. In conclusion, miR-155 might serve as a new tumor biomarker in the clinicopathological diagnosis and prognostic assessment in CRC.

MiR-224 expressed in primary tumor samples functions as a pro-metastatic rather than anti-metastatic in CRC partly via the regulation of SMAD4 [50]. MiR-224 is the most up-regulated miR in inflammatory bowel disease (IBD)-associated CRC by targeting p21 [51]. Elevation of miR-224 in CRC directly targeted 3’-UTRs of tumor suppressors PH domain leucine-rich-repeats protein phosphatase 1 (PHLPP1) and PHLPP2 to promote the growth and tumorigenicity in CRC [52]. Moreover, the abnormal expression of miR-224 up-regulated glycogen synthase kinase 3 beta (GSK-3β), inhibited Wnt/β-catenin signal pathway and reduced adramycin (ADM) resistance to CRC [53]. The high expression level of miR-224 connected with poor disease-free survival rate partially through suppression of SMAD4 that function as transmitters of TGF-β signals, and decreases the chemoradiosensitivity (CRT) in CRC, highlighting the function of miR-224 in the process of potential predictive marker for relapse following radical surgery of colorectal cancer [54].

In addition, other panels of miRNAs were identified as being associated with the pathogenesis of CRC. For instance, higher level of signal transducer and activator of transcription 3 (STAT3)-mediated transcription of miR-214 was associated with ulcerative colitis (UC) and progression to colitis-associated colon cancer (CAC), which induced by interleukin-6 (IL-6), increases phosphorylation of AKT, and activates NF-κB through direct repression of PTEN, PDZ and LIM domain 2 (PDLIM2) [55]. MiR-31 has been shown to improve colorectal cancer cell growth and stimulating tumorigenesis by directly recognizing the specific location within the 3′-UTR of RAS p21 GTPase activating protein 1 (RASA1) transcripts [56]. MiR-210, a hypoxia-inducible miRNA, is expressed in a wide range of cells and is involved in cell proliferation, mitochondrial respiration, DNA repair, vascular biology, and angiogenesis. In CRC, the overexpression correlates well with poor prognosis and up-regulated levels of miR-210 stimulate angiogenesis by enhancing RNA Binding Motif Protein 3 (RBM3) expression [57]. MiR-182 and miR-503 undergo sequential up-regulation and contribute to the malignant transformation of colon adenoma to adenocarcinoma by cooperatively down-regulating the tumor suppressor F-Box and WD repeat domain containing 7 (FBXW7) which is the substrate recognition component of a ubiquitin ligase complex involved in cell cycle and targeted proto-oncogenes such as c-Myc and cyclin E2 [58]. MiR-200c has also been shown to function as an oncogene in CRC played an important role in regulation of EMT and metastatic behavior in CRC results in the negative regulation of its gene targets zinc finger E-box binding homeobox 1 (ZEB1), ETS proto-oncogene 1, transcription factor (ETS1) and fms related tyrosine kinase 1 (FLT1), which, in turn, regulates the EMT markers (E-cadherin and vimentin) [59]. Furthermore, the silencing of miR-200c also leads to up-regulation of the PTEN and p53 tumor suppressor genes [57]. Moreover, another oncogenic miR-301a was significantly higher in lymph node metastasis positive CRC samples involved in regulation of migration and invasion by targeting the downstream gene TGFBR2 or NF-κB/STAT3 to promote tumorigenesis [60]. Taken together, a great deal of miRNAs exerted their functions as oncogene through multiple targets (Table 1), and can be served as a potential diagnostic marker and therapeutic target for CRC patients.

### 3.2. Tumor-Suppressive miRNAs in CRC

Tumor-suppressive miRNAs play an important role in retarding tumor progression through down-regulating oncogenes associated with proliferation, apoptosis, invasion, and migration. An overview of the studies investigating the role of current tumor-suppressive miRNAs associated with CRC development is depicted in Table 2.

Lethal-7 (let-7) family consists of 12 miRNA members (let-7a-1/2/3, -7b, -7c, -7d, -7e, -7f-1,/2, -7g, -7i, and miR-98), is highly conserved in different species and take part in the EMT process, by targeting oncogenes such as RAS, c-Myc, cell division cycle 34 (CDC34), CDC25A, cyclin dependent kinase 6 (CDK6), high mobility group AT-hook 2 (HMGA2), lin-28 homolog (LIN28) and LIN28B [61]. In colon cancer tissues, let-7 was remarkably down-regulated compared to the adjacent noncancerous tissues. Further studies in clinical specimens have shown that let-7 suppressed RAS after binding to the complementary 3′-UTR of the kirsten rat sarcoma viral oncogene homolog (KRAS) mRNA, mainly by suppressing the mutation of KRAS under EGFR-targeted clinic treatment. Intra-tumor let-7a expression was significantly associated with better survival outcomes in CRC patients [62]. In addition, the cluster miR-99a/let-7c/miR-125b was identified to play an important role in regulating CRC patient response to anti-EGFR targeting therapy, suggesting that miR-99a/let-7c/miR-125b signature could improve the selection of patients with KRAS wild-type CRC as good candidates for anti-EGFR therapy [63]. Moreover, Let-7g may act as a potential tumor-suppressor and associated with clinical outcomes of colorectal cancer [64]. These data revealed that let-7 family is a key tumor-suppressor in CRC therapy.

MiR-194 was frequently decreased in CRC, with this level closely associated with the overall survival of patients and obviously associated with tumor size, as well as the tumor node metastasis (TNM) stage that is involved with cell proliferation, colony formation, promoted G0/G1 arrest, and induced cell apoptosis through negative regulation of the MAP4K4/c-Jun/MDM2 or PDK1/AKT2/XIAP signaling pathway, and acts as an tumor inhibitor in CRC, but not related to lymphatic invasion and distant metastasis [65,66]. In addition, the low expression levels of miR-194 in patients, correlated with advanced colorectal adenoma (ACRA) after polypectomy, used independently as a better predictor for adenoma recurrence [67]. MiR-194 target genes display concordant expression profiles with oncogenic transcriptional regulator high-mobility group AT-hook 2 (HMGA2) involved in limiting cell proliferation and migration, reducing xenograft growth, attenuating cell migration, suppressing EMT, and promoting anticancer drug sensitivity in CRC [68].

The miR-143 and miR-145, closely located in a 1.6kb cluster with 5q32-33 chromosomal region, are frequently displayed as coordinated expression profile from a single promoter suggesting they originate from the same primary transcript containing both miRNAs [69]. The expression of both miRNAs have been shown to be down-regulated in several cancer types, including CRC, with the function of anti-tumorigenic activity involved in altering various cancer-related cellular processes that include: proliferation, invasion, migration, chemo-resistance, growth in an anchorage-independent manner, and undergo apoptosis on genotoxic stimulation [70]. Most studies on miR-143 and miR-145 were independent and did not consider them as concomitant re-expression genes, while many reports showed them as interdependent genes in their down-regulated cancer cell lines. Michael et al. first identified the association between miRNAs and CRC via uncovering the down-regulation of miR-143 and miR-145 in CRC tissues compared to the normal mucosa specimens [71]. MiR-143 and miR-145 target specific sites of insulin-like growth factor 1 receptor (IGF1R) by directly recognizing the 3′-UTR of the IGF1R transcript and regulates IGF1R expression [72]. Dysregulated miR-143-145 clusters may be functionally associated with the pathogenesis of synchronous CRC. Polymorphism rs4705341 and rs353292 in the flanking region of miR-143 and miR-145 was associated with CRC risk and clinical outcome, especially the rs4705341 GG genotype [73,74]. The polymorphisms in the promoter region of these miRNAs were also reported to link with the etiology of CRC [75]. Pagliuca revealed that the gene cluster miR-143 and miR-145 were diminished and played a coordinated shared either with the target transcript, or the common signaling pathway by targeting cluster of differentiation 44 (CD44), Kruppel-like factor 5 (KLF5), KRAS and v-Raf murine sarcoma viral oncogene homolog B1 (BRAF) belonging to the growth factor receptor–mitogen-activated protein kinase network and the p53 signaling pathway [76]. Moreover, stable miR-143 or miR-145 overexpression increased cell sensitivity to cetuximab-mediated antibody-dependent cellular cytotoxicity (ADCC) and apoptosis, resulting in a significant increase of cetuximab-mediated ADCC independent of KRAS status [77]. In conclusion, miR-143 and miR-145 belong to the family of oncosuppressive miRNAs, thus, may serve as therapeutic targets for cancer intervention.

MiR-34a is located on chromosome 1p36.23, belongs to the miR-34 family (miR-34a/b/c). MiR-34a has been identified to be down-regulated in various types of tumors, including CRC, as an important tumor suppressor gene [78]. Recently, the aberrant CpG promoter methylation with miR-34a silencing was found in CRC, via inactivating or weakening the checkpoints by p53 [79]. Moreover, the single nucleotide polymorphism (SNP) located in the mature region of miR-34a was highly associated with a decreased risk of CRC by regulation of 3’-UTR in tumor-promoting gene E2F transcription factor 1 (E2F1) [80]. In addition, there is a positive feedback mechanism between miR-34a and p53, which induced expression of miR-34a that inhibited the expression of silent information regulator 1 (SIRT1), to up-regulate the acetylation and transcriptional activity of p53 and expression of p21/PUMA. Thus, miR-34a functions as a tumor suppressor to regulate cellular growth, in part, through a SIRT1-p53 pathway [81]. MiR-34a also behaves as a tumor suppressor in CRC by inhibiting cancer cell proliferation, invasiveness and metastasis through the down-regulation of formin like 2 (FMNL2) and E2F transcription factor 5 (E2F5) expressions [82]. Furthermore, lncRNA small nucleolar RNA host gene 7 (SNHG7) facilitated the proliferation and metastasis through regulating the PI3K/Akt/mTOR axis to increase the expression of polypeptide *N*-acetylgalactosaminyltransferase 7 (GALNT7), and miR-34a contributed to the progression of CRC by targeting lncRNA SNHG7 [83]. MiR-34a also mediated oxaliplatin chemo-resistance through its inhibitory effects on macroautophagy via regulation of the transforming growth factor-β/Smad4 pathway [84]. Regorafenib-mediated suppression of colon tumorigenesis was associated with the increased level of miR-34a, resulting in reversing drug resistance by degrading Wnt/β-catenin in CRC [85]. MiR-34a combined with 5-FU can increase the rate of cell apoptosis to inhibit cell growth, which suggested that miR-34a might increase the sensitivity of colorectal cancer cells to 5-FU, function as a predictor of fluorouracil chemosensitivity, as well as be expected to be more beneficial to patients with CRC [86].

MiR-126 is located on chromosome 9q34.3, within the 7th intron of epidermal growth factor-like domain 7 (EGFL7), and contributes to progression of angiogenesis, proliferation, migration, invasion, and cell survival via inactivation of the oncogene signaling pathway. Patients with low expression of miR-126 showed poor prognostic outcome [87]. The expression of miR-126 was significantly reduced, particularly in highly metastatic CRC cell lines, and the up-regulated expression of miR-126 inhibited the growth of CRC cells [88]. MiR-126 suppressed the growth of CRC neoplastic partly by targeting the p85β subunit of phosphatidylinositol 3-kinase (PI3K) which is highly correlated to cell survival and carcinogenesis through phosphorylation of downstream effectors, such as NF-κB and mechanistic target of rapamycin kinase (mTOR) [89]. Moreover, miR126 inhibited the expression of vascular cell adhesion molecule-1 (VCAM-1), which led to carcinogenic transformation, angiogenesis, and metastasis in CRC [90]. MiR-126 also targeted the 3′-UTR of C-X-C motif chemokine receptor 4 (CXCR4) directly to suppress CRC cell viability, migration, and invasion capacity by invading through the vascular basement membrane, which is associated with endothelial cell growth, angiogenesis, and hematopoiesis [91]. The decreased expression of miR-126 is associated with enhanced angiogenesis by targeting vascular endothelial growth factor A (VEGFA) and enhanced cell proliferation, migration and invasion [92]. In addition, over-expression of endogenous miR-126 and exogenous miR-126 directly mimic targeting the IRS-1/AKT/ERK or RhoA/ROCK signaling pathways, as well as inhibited CRC cells’ proliferation, migration, and invasion, which resulted in cell cycle arrest, but had no effect on cell apoptosis [93,94]. More specifically, studies have shown the expression of serum miR-126 was down-regulated in synchronous liver-metastatic CRC (SLM-CRC) and organ-metastatic CRC (OM-CRC) supporting miR-126 as a novel biomarker for earlier detection or clinical diagnosis of early-stage liver metastasis from CRC.

MiR-27b, which is located on chromosome 9q22.32, suppresses neuroblastoma cells via targeting the peroxisome proliferator-activated receptor γ (PPARγ), acts as an angiogenic switch in control of the endothelial tip cell fate and sprouting [95,96]. However, the information about miR-27b in CRC remains scarce. VEGF as a functional downstream target of miR-27b involved in blocking CRC cell proliferation, colony formation, tumor growth, and angiogenesis inhibition, where miR-27b-mediated gene silencing in CRC was attributable to reversible DNA hypermethylation of the CpG islands and not histone acetylation [97]. Further studies suggested that miR-27b inhibited CRC cell growth and invasion through targeting Rab3D (a member of the Ras superfamily of monomeric G proteins), and promoted metastasis through activating Akt/GSK3β/Snail pathway and inducing the EMT process [98]. MiR-27b expression is tightly associated with tumor size, TNM stage, and overall survival time in CRC patients. 

Additionally, there are other panels of miRNAs that function as tumor-suppressive in CRC. For instance, miR-7 was down-regulated in CRC specimens and cell lines by targeting EGFR and raf-1 proto-oncogene (RAF-1), a gene downstream of KRAS to inhibit translation and potently suppressed the proliferation of CRC [99]. MiR-18a-3p (also termed microRNA18 passenger strands, miR-18*) directly and specifically targeted the KRAS oncogene to suppress proliferation and anchorage-independent growth in colon adenocarcinoma HT-29 cells, serving as the potential target or therapeutic agent for cancer therapy [100]. Moreover, the overexpression of miR-26b was reported to target four different oncogenes: TATA-box binding protein associated factor 12 (TAF12), protein tyrosine phosphatase type IVA, member 1 (PTP4A1), checkpoint with forkhead and ring finger domains (CHFR), and STE20-related kinase adaptor beta (ALS2CR2), to suppress the cell growth and the induction of apoptosis associated with the invasiveness and metastasis of CRC cells. MiR-26b targets the 3′-UTR of fucosyltransferase4 (FUT4) affected migratory behavior of CRC cells to inhibit cell aggressiveness and down-regulated sphingosine kinase 1 (SphK1) mRNA and protein expression to inhibit the growth of CRC cells and increase the sensitivity of chemo-resistance and other cancerous behaviors [101,102]. MiR-101 confers a role as a tumor suppressor via direct inhibition of prostaglandin-endoperoxide synthase 2 (COX-2) mRNA translation at the post-transcriptional level in CRC [103], or directly targeting ZEB1 to inhibit the proliferation and migration of CRC cells [104]. In addition, there were many other miRNAs, such as miR-144 [105], miR-320a [106], miR-330 [107], miR-455 [108], and miR-149 [109], that function as a tumor suppressor involved in apoptosis, migration, proliferation, invasion, and cell cycle single pathways by targeting different oncogenes. In summary, these studies identified various tumor-suppressive miRNAs (Table 2), suggesting the application of miRNAs in prognosis prediction and cancer treatment.

## 4. Conclusions

CRC is one of the most common cancer which is a leading cause of cancer-related morbidity and mortality. The association between miRNAs and CRC was first identified by Michael et al. in 2003 [69]. Substantial progress has revealed the relationship between miRNAs and CRC, where miRNAs were proved to participate in multiple biological processes, such as cell cycle, apoptosis, invasion, migration, and metastasis in CRC. As important regulators of gene expression, miRNAs provide an additional level of control of gene expression, largely at the posttranscriptional level. Sustained dysregulation of miRNAs play vital roles in the development of CRC by detecting the targets and downstream regulatory molecules in a context-dependent manner, acting as oncogenes or tumor suppressors. In this review, we focused on several microRNAs shown to be specific to CRC, their roles in CRC biology was summarized in association with molecular processes underlying carcinogenesis and progression of CRC. Although the precise mechanisms involved in carcinogenesis are not yet defined, the investigative studies supplied a better understanding of CRC-related miRNA functions and their roles as tumor suppressors, as well as oncogenes involved in CRC development, invasiveness, prognosis, and treatment response. On the other hand, many studies indicated that abnormally expressed miRNAs in CRC often associated with the clinical signatures that usually correlated with CRC progression or metastasis, which could open new horizons of a novel opportunity to develop new ways of understanding the disease mechanism and potential diagnostic and/or prognostic markers. Ongoing research geared towards understanding miRNA biology, delivery system innovations, and detection methodologies will lead to better knowledge of the relationship between CRC and microRNAs, which will potentially change the CRC diagnosis and treatment, to produce potentially beneficial agents for the prognosis and therapy of CRC, thus direct medicine into a new era of effectiveness.

## Figures and Tables

**Table 1:** The up-regulated oncomiRs in CRC.

MiRNA	Direct/Indirect Targets	Functions	Reference
MiR-21	PDCD4, TIAM1, SPRY2, PTEN, TGFBR2, CDC25A, hMSH2	Proliferation, Apoptosis, Invasion, Migration, CSC maintenance, Intravasation, Cell cycle, Chemo-resistance	[23,24,25,26,27]
MiR-92a	PTEN, SMAD2, SMAD4, TGFBR2	EMT, Invasion, Venous invasion, Metastases, Proliferation	[29,30]
MiR-96	TP53INP1, FOXO1, FOXO3A, UBE2N, XIAP, REV1, RAD51	Cell growth, Proliferation, Drug-sensitizing, Apoptosis	[32,33,34]
MiR-135a/b	APC, hMLH1, hMSH2	Proliferation, Gene dosage effects	[37,38]
MiR-155	PTPRJ, TP53INP1, MSH2, MSH6, MLH1, FOXO3a, HuR	Proliferation, Invasion, Stemness, Angiogenesis, Drug resistance, Genome instability, ETM	[42,43,44,45,46,47,48,49]
MiR-224	SMAD4, p21, PHLPP1, PHLPP2, GSK-3β	Metastasis, Proliferation, tumorigenicity, Chemoradiosensitivity	[50,51,52,53,54]
MiR-214	PTEN, PDLIM2	Inflammation	[55]
MiR-31	RASA1	Proliferation	[56]
MiR-210	RBM3	Proliferation, Mitochondrial respiration, DNA repair, Vascular biology, Angiogenesis	[57]
MiR-182/503	FBXW7	Malignant transformation	[58]
MiR-200c	ZEB1, ETS1, FLT1, EMT markers (E-cadherin, vimentin), PTEN	Metastatic, Proliferation, Invasion, Migration	[57,59]
MiR-301a	TGFBR2	Lymph node metastasis, Migration, Invasion	[60]

**Table 2:** The down-regulated tumor-suppressive miRNAs in CRC.

MiRNA	Direct/Indirect Targets	Functions	Reference
Let-7	KRAS	Proliferation	[62,63]
MiR-194	MAP4K4, AKT2	Proliferation, apoptosis, invasion, migration, cell cycle	[65,66]
MiR-143/145	IGF1R, CD44, KLF5, KRAS, BRAF	Proliferation, invasion, migration, apoptosis, angiogenesis, chemo-resistance,	[72,76]
MiR-34a	E2F1, SIRT1, FMNL2, E2F5, SNHG7	Proliferation, invasiveness, metastasis, apoptosis, chemo-resistance	[80,81,82]
MiR-126	PI3K, VCAM-1, CXCR4, VEGFA, IRS1, RhoA	Proliferation, invasion, migration, cell cycle, angiogenesis, hematopoiesis	[89,90,91,92]
MiR-27b	VEGF, Rab3D	Proliferation, colony formation, angiogenesis, EMT	[97,98]
MiR-7	EGFR, RAF-1	proliferation	[99]
MiR-18a-3p	KRAS	Proliferation, anchorage-independent growth	[100]
MiR-26b	TAF12, PTP4A1, CHFR, ALS2CR2, FUT4	Proliferation, apoptosis, invasiveness, metastasis, migration, chemo-resistance	[101,102]
MiR-101	COX-2, ZEB1	Proliferation, migration	[103,104]
MiR-144	mTOR	Proliferation	[105]
MiR-320a	β-catenin	Proliferation	[106]
MiR-330	CDC42	Proliferation	[107]
MiR-455	RAF1	Proliferation, invasion	[108]
MiR-149	FOXM1	Proliferation, migration, invasion	[109]
